# Modern Plant Breeding Techniques in Crop Improvement and Genetic Diversity: From Molecular Markers and Gene Editing to Artificial Intelligence—A Critical Review

**DOI:** 10.3390/plants13192676

**Published:** 2024-09-24

**Authors:** Lixia Sun, Mingyu Lai, Fozia Ghouri, Muhammad Amjad Nawaz, Fawad Ali, Faheem Shehzad Baloch, Muhammad Azhar Nadeem, Muhammad Aasim, Muhammad Qasim Shahid

**Affiliations:** 1 State Key Laboratory for Conservation and Utilization of Subtropical Agro-Bioresources, Guangdong Laboratory for Lingnan Modern Agriculture, South China Agricultural University, Guangzhou 510642, China; alicia2222@stu.scau.edu.cn (L.S.); mylai@stu.scau.edu.cn (M.L.); foziaghouri@scau.edu.cn (F.G.); 2 Guangdong Provincial Key Laboratory of Plant Molecular Breeding, South China Agricultural University, Guangzhou 510642, China; 3 Education Scientific Center of Nanotechnology, Far Eastern Federal University, 690091 Vladivostok, Russia; m.a.nawaz.cabb.uaf@gmail.com; 4 School of Tropical Agriculture and Forestry, Hainan University, Sanya 572025, China; fawadali365@gmail.com; 5 Dapartment of Biotechnology, Faculty of Science, Mersin University, Mersin 33343, Türkiye; balochfaheem13@gmail.com; 6 Faculty of Agricultural Sciences and Technologies, Sivas University of Science and Technology, Sivas 58140, Türkiye; azharjoiya22@gmail.com (M.A.N.); mshazim@gmail.com (M.A.)

**Keywords:** CRISPR/Cas9, DNA molecular markers, gene regulation, GWAS, MAS, QTL mapping

## Abstract

With the development of new technologies in recent years, researchers have made significant progress in crop breeding. Modern breeding differs from traditional breeding because of great changes in technical means and breeding concepts. Whereas traditional breeding initially focused on high yields, modern breeding focuses on breeding orientations based on different crops’ audiences or by-products. The process of modern breeding starts from the creation of material populations, which can be constructed by natural mutagenesis, chemical mutagenesis, physical mutagenesis transfer DNA (T-DNA), Tos17 (endogenous retrotransposon), etc. Then, gene function can be mined through QTL mapping, Bulked-segregant analysis (BSA), Genome-wide association studies (GWASs), RNA interference (RNAi), and gene editing. Then, at the transcriptional, post-transcriptional, and translational levels, the functions of genes are described in terms of post-translational aspects. This article mainly discusses the application of the above modern scientific and technological methods of breeding and the advantages and limitations of crop breeding and diversity. In particular, the development of gene editing technology has contributed to modern breeding research.

## 1. Introduction

The history of crop breeding dates back to the time when humans started cultivation, selection, and harvesting. From then on, crop breeding has become a discipline guided by principles and breeding techniques consisting of theoretical and practical skills and knowledge. Major developments took place during the past 1000 years, where humans have been using artificial selection to select and breed plants with higher nutritional value [1]. Since Mendel’s laws were re-emphasized, breeders began to use systematic breeding by screening the excellent variant varieties in the existing domesticated variety groups and constantly investigating the varieties’ various agronomic traits to improve the varieties’ yield and quality. Over time, population improvement and recurrent selective breeding [2] have gradually entered the breeder’s field of vision.

**Figure 1:**
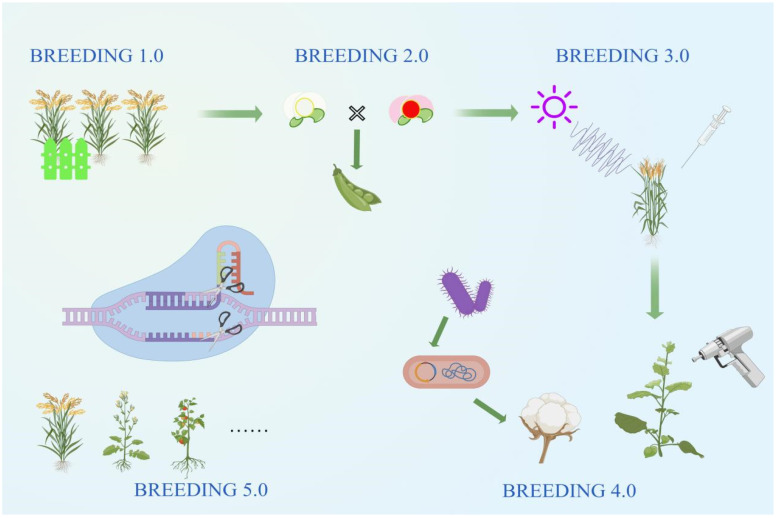
Landmark stages of plant breeding. Breeding 1.0 is the use of human selection and the breeding of crops with higher nutritional value. Breeding 2.0 involves the use of human-mediated cross-breeding and the application of Mendelian genetics principles. Breeding 3.0 involves the use of physical and chemical mutagenesis. Breeding 4.0 involves the use of genetic engineering. Breeding 5.0 involves the use of gene editing technology. The figure was generated in Figdraw (www.figdraw.com).

In the past, traditional agricultural methods were the only factor contributing to diverse genetic material for food, cash, feed, and medicinal crops. However, recent developments in crop breeding and genomics have provided further opportunities in plant breeding. Plant breeders have proposed different ways to divide the historical developments in crop breeding. For example, Professor Buckler [3], a member of the American Academy of Sciences, proposed the concept of “Breeding 4.0” and showed that the development of crop breeding technology has already gone through three landmark stages. Other plant breeders, such as Liu et al. [4], have divided the history of the developments in plant breeding into five stages (Figure 1).

The main food and feed crops are rice, wheat, maize, sugar cane, potatoes, and soybean [5]. Their breeding methods can be broadly divided into conventional and non-conventional breeding [6]. Crossbreeding and distant hybridization belonging to conventional breeding are relatively traditional, while non-conventional breeding includes mutation breeding, in vitro culture, and molecular breeding. These non-conventional breeding methods, such as molecular breeding, e.g., gene editing, are relatively fast and efficient breeding methods [7,8]. On the contrary, conventional plant breeding methods include domestication, selection, and crossing/hybridization. Several distinct methods have been developed for breeding self- and cross-pollinated crop plants and vegetatively propagated plants. For a detailed understanding of the breeding methods for individual crop plants, the readers are referred to the articles by Yuan, Whitford [9], Schnable [10], and Neele [11].

Traditional breeding has always been proven to be an indispensable link between the availability of natural variation and the development of new high-yielding cultivars. However, it mainly involves the improvement of agronomic traits on the basis of phenotypic selection. A drawback of such type of selection is that if the trait does not meet the breeder’s breeding objective, it will be neglected [12]. Although several traits have been improved through selective breeding, it has also caused the non-selection of important genes associated with agronomic and non-agronomic traits of interest. Continued phenotypic selection has narrowed down the available genetic variation. Since variability is the key requirement of a plant breeding program, plant breeders realized that the creation and search for novel sources of variation are critical, such as induced-mutation breeding [13], ploidy breeding [14,15], genetic engineering [16], chromosome engineering [17], molecular marker breeding [18], etc. In order to study variation, plant breeders have to understand and process large-scale phenotypic data, which makes it difficult to recognize the relationship between genes and phenotypes. Modern techniques such as quantitative trait loci (QTL) mapping have enabled plant breeders to create and explore a population to study the function of genes [19]. Gene mapping can be realized by establishing backcross population (BC), filial generation (F_2_), double haploids (DH), near-isogenic lines (NILs), and recombinant inbred lines (RILs) using genetic markers and gene mapping [20,21,22,23]. It is also possible to select individuals with significantly different target shapes for mixing pools and use BSA (Bulk Segregation Analysis) technology for analysis [24]. Genome-wide association study (GWAS) is a crucial method for discovering genes linked to agronomic traits in crop species to improve crop yields effectively [25,26]. Even though GWAS has been extensively utilized in breeding to increase yield, single-trait analysis has been the main focus of most studies. Single-trait GWAS has restrictions that may prevent certain significant pleiotropic SNPs (pSNPs) from being found for complex characteristics, like rice yield, which is influenced by many factors. This limits the ability to delve further into the genetic basis of complex traits. Multi-trait has demonstrated their greater ability to understand intricate factors and enhance their effectiveness in identifying pleiotropic associations by considering correlations among multiple traits [27]. Many multi-trait GWAS techniques are currently available that can be used with plants, including MTMM [28], PC-GWAS [29], TATES [30], and mvLMM [31]. These techniques easily detect pleiotropic loci and effectively limit false positives resulting from population structure. Nevertheless, many of these techniques prioritize increasing detection speed and power while frequently ignoring how pleiotropic loci can be interpreted. The study of the pangenome can show the diversity of species and dig out genes related to important traits in multiple varieties of the same species [32]. Therefore, the new breeding technologies present tremendous opportunities for crop improvement and increased production [33,34].

Further developments in the field of genetics and genomics have made it easier to study and understand the gene functions associated with specific traits. According to the central dogma of molecular biology, the process of genetic information transmission is not static, and each link has its own response mechanism. For example, in the study of super hybrid rice by using high-throughput sequencing, it was found that small RNAs are an important factor in regulating its heterosis [35]. In Arabidopsis, the epigenetic modification of the biological clock gene CCA1 (CIRCUIT CLOCK ASSOCIATED 1), the extended hypocotyl gene LHY (LATE ELONGATED HYPOCOTYL) genes, their mutual regulatory factors TOC1 (TIMING OF CAB EXPRESSION 1), and GI (GIGANTEA) mediate downstream gene and pathway expression changes, thereby regulating the heterosis [36]. Heterosis is usually associated with transcriptional variation between inbred lines and hybrids [37,38]. Transcriptome analysis of Arabidopsis hybrids revealed changes in the expression of defense and stress-response genes consistent with a decrease in basal defense levels, indicating that this hormone-regulated defense and stress response was conducive to the heterosis of Arabidopsis [39]. Therefore, the combination of conventional breeding methods and molecular-level research has a higher efficiency in improving crop yield and quality.

Although plant breeders have developed conventional and unconventional breeding methods, some conventional techniques are no longer suitable for today’s efficient and fast breeding cadence. Therefore, it is important to break through these shortcomings. To make matters worse, in the process of breeding, we still lack supporting cultivation techniques as we still lack a mature application system of breeding techniques [40,41,42]. Therefore, breeders need to solve or improve these key issues. This paper discusses common breeding methods and identification techniques, summarizes their applications in crop breeding, and highlights their existing problems. Thus, this review intends to stress the need for accelerated research on developing new breeding methods.

## 2. Germplasm Enhancement and Genetic Diversity Creation

Plant breeding has had a significant impact on food production in order to ensure world food security. However, it has also brought about agricultural homogeneity throughout farm fields, which puts crops at risk from biotic and abiotic pressures due to reduced genetic variability. The emergence of epidemics like the American maize blight in the 1970s and the Irish potato blight in the 1840s has provided ample evidence of these risks [43]. Genetically uniform wheat is also under threat from highly pathogenic novel stem rust Ug99 race from East Africa [44]. Hence, it is imperative to devise practical approaches for achieving sustainable agriculture by finding the proper equilibrium between maximizing crop yield in specific circumstances and reducing the vulnerability to crop failure when conditions alter. To achieve such a balance, more knowledge on how modern plant breeding affects agricultural genetic diversity is needed [45]. The comparatively un-directed process of evolution was the only source of variation to the domestication of plants and animals in earlier times. Furthermore, conventional breeding methods are insufficient for enhancing the plant genome. Therefore, a targeted drive to produce the desired variation can be made possible only when implementing modern plant breeding technologies to overcome this challenge.

A sufficient amount of genetic diversity can not be created using conventional breeding methods alone. Modern plant breeding strategies have been developed to address this hurdle in plant breeding practices. Modern plant breeding techniques emerged when molecular methods and other cutting-edge approaches were combined with traditional breeding strategies or introduced separately to produce crop genetic variety. This was accomplished by determining the genotypes and phenotypes of the crop’s desirable traits. Modern plant breeding techniques such as marker-assisted selection, high throughput phenotyping, genome editing, genetic mapping, epigenetic modifications, transcriptional and translational regulation, mutation, and artificial intelligence were used to create crop genetic diversity.

Humans have a long history of exploiting natural variation through the domestication of wild germplasm into cultivated crops and hybridization [5]. One of the major sources of natural variations is spontaneous mutations. They occur in organisms without any artificial treatment but under the influence of natural conditions. Crops that arise from natural mutations include rice cultivars Dwarf Nantes, glutinous maize [46], and sweet corn [47]. However, low frequency, limited valuable variation, and low efficiency in generating new germplasm have shifted breeders’ attention to unconventional breeding. This gave rise to several mutation breeding methods, i.e., physical and chemical mutagenesis, T-DNA insertion, and Tos17 transposon mutagenesis. After studying and exploiting these mutants, breeders have created a large population of excellent germplasm resources, e.g., Ethyl Methanesulfonate (EMS)-induced mutation resources have been created in maize comprising 2050 independent M2 mutant families in the elite tropical maize inbred ML10 [48], a TILLING population in B73 comprising 750 M1 plants [49], an indexed population in B73 that has been exome-sequenced comprising 1086 M1 plants [50,51]

### 2.1. Physical Mutagenesis

Physical mutagenesis uses radiation as mutagens, such as α-rays, β-rays, γ-rays, ultraviolet radiation, microwave radiation, and other physical factors, to induce plant mutations. Radiation can cause chromosome breaks and reconnection, resulting in mutations in chromosome structure and number as well as changes in bases in the structure of DNA molecules [52]. Genetic variation occurs when these mutant individuals reproduce and transfer the mutated genetic material to sexual cells or asexual reproductive organs.

In 1928, Stadler [53] pioneered the physical mutagenesis of plants by mutagenizing corn and barley with X-rays. Physical mutagenesis has been mainly used to improve seed-propagated crops such as rice [54], barley [55], and cotton [56]. Since the advent of X-rays about a hundred years ago, ionizing radiation has generated significant genetic diversity for crop improvement [57]. Methods of using physical mutagens are well established now. As of 2024, more than 1,700 mutant varieties involving 154 plant species have been released in 50 countries worldwide [58,59]. Oladosu [60] summarized many successful cases of new rice varieties developed by radiation-induced mutagenesis. Previously, it was reported that mutagenesis affected plant height and tillering in rice plants [61], resulting in significant changes both in DNA methylation and gene expression [62]. This special and complex environment brings new opportunities for mutation induction, and space breeding will be an important tool for breeding new crop varieties in the future [63].

Though physical mutagenesis is a favorable breeding method for crop improvement, it comes with several drawbacks. The crucial aspect is that physically produced mutations are inherently random, necessitating extensive screening efforts. Mutagenesis often results in more than excellent agronomic traits. Therefore, breeders must also use genetic methods to cross and screen generations to eliminate unfavorable mutations [64].

### 2.2. Chemical Mutagenesis

Chemical mutagenesis refers to the treatment of crop plants with chemical mutagens to induce mutations [65]. Chemical mutation enables plants to develop new traits that are unavailable in nature or difficult to obtain by conventional methods [66]. At the same time, it is not questioned by risk assessment agencies in terms of safety, like the development of transgenics [67]. Commonly used chemical mutagens include alkylating agents, base analogs, antibiotics, azides, nitriles, hydroxylamines, and acridines (reference). Ethyl Methanesulfonate (EMS) is an alkylating agent that causes base mismatches and substitutions through the conversion of purines to pyrimidines [68]. Compared with other mutagens, EMS mutagenesis produces a high frequency of mutations, and most of them are dominant mutants, which are easy to screen for mutants. Therefore, EMS is currently the most widely used mutagen in crop mutation breeding [69]. 

The technique of chemical mutagenesis has proven to be effective in the selective breeding of various important crops. For example, Johanna [70] et al. developed a mutant population using EMS-inducible wheat and screened for new salt-tolerant wheat varieties, which are beneficial for saline agriculture. Similarly, Yu [71] et al. used EMS as a mutagen and constructed a mutant collection containing 3223 bitter melons, which not only brought new germplasm resources for bitter melon breeding but also accelerated the study of its gene functions. It has also been shown that sugarcane mutants screened by EMS induction showed improvements in agronomic traits such as resistance to black spike disease, early maturity, and high yield [72]. 

Chemical mutagenesis, like physical mutagenesis, is a method developed by breeders to accelerate the rate of mutation and thus accelerate breeding artificially. It is similar to physical mutagenesis; due to the uncertainty of mutation, the direction and nature of mutagenesis cannot be controlled, and it has blindness. In addition, it is difficult to chemically induce and identify micromutations in quantitative traits.

### 2.3. T-DNA Insertion

It has been discovered that the Ti plasmid contains a distinct DNA fragment [73], which is randomly integrated into the plant genome during the transformation process. The insertion of exons or introns often leads to gene inactivation, and different genomic integration sites result in diverse gene mutations, leading to mutant phenotypes. Upon Agrobacterium infection of plant cells, this fragment can be spontaneously transferred and inserted into the plant chromosome DNA, which is henceforth referred to as T-DNA [74]. Breeders found that T-DNA carrying foreign genes can be used to infect plants, which can cross the nuclear membrane into the nucleus and randomly integrate into the nuclear genome, and the T-DNA integrated into the nuclear genome can be inherited stably [75]. Therefore, T-DNA-mediated transformation has also become a common method for generating new plant varieties. In addition, T-DNA insertion mutation also plays a key role in forward and reverse genetics research [76]. T-DNA tags have helped us identify a large population of mutants [77] and various genes associated with important functions [78,79,80]. As a model plant for dicotyledons, Arabidopsis is commonly used as a material for T-DNA insertional mutations [81], and its mutants are a valuable resource for exploring gene function and elucidating metabolic and signaling pathways. The T-DNA rice lines developed by Jeon [82] et al. can help identify insertional mutations in various genes and can be used to discover novel genes in rice. The widespread use and emphasis on T-DNA insertion mutation has made it one of the most commonly used induction techniques for plant germplasm innovation and has led to the development and growth of this technology. 

Contrary to this, Gao and Zhao [83] found that some Arabidopsis T-DNA mutations are stably suppressed by T-DNA inserted into other non-homologous loci. Studies indicate that T-DNA insertion structures can affect hosts’ epigenetics and negatively impact transgenic function [84]. These results suggest that T-DNA insertion mutations need to be used with caution. Moreover, not all species have established complete and efficient transformation systems, and the transformation process is time-consuming and costly, which is also an urgent problem to be solved in building mutant libraries.

### 2.4. Transposable Elements as a Source of Variation

A transposon is a kind of DNA sequence that can move autonomously on the genome. It was first discovered in maize by cytogeneticist McClintock in 1938. When transposon is inserted into a functional gene, it may cause inactivation of the gene, thus inducing mutation. Transposons can also provide homologous sequences to promote homologous recombination, can be inserted into new sites after reverse transcription, and the trans-factor or cis-sequence encoded by them can also cause gene rearrangement [85]. Successful examples of using transposon-based mutation breeding include the identification of 32 retrotransposons in rice. Of these, Tos17 is an active endogenous retrotransposon, and its activity is mainly regulated at the transcription level [86]. Tos17 can actively move and induce plant mutation when activated during tissue culture [87]. Therefore, transposon insertion is an important technology for breeders to obtain a large number of rice mutants at present [85]. It has been shown that the Tos17 insertion gene increased protein and amino acid content in the mutant, suggesting that it may have the much-needed potential for improving rice quality [88]. Kyong [89] et al. used two local varieties, “Ilmibyeo” (IM) and “Baegjinh1ho” (BJJ1), in their experiments to not only develop new insertion mutants but also to demonstrate the potential utility of the Tos17 mutant to improve agronomic traits in various rice varieties through effective tissue culture methods. The Tos17 system can also significantly contribute to the functional genomics of rice and provide strong technical support for the development of new germplasm resources in rice [90].

However, the Tos17 mutant library has a large number of copies inserted, which is inconvenient for genetic analysis in the future. Moreover, only 10% of the resulting mutants are truly caused by Tos17 insertion, while the remaining mutants are produced in tissue culture or by other unknown transposons. A research team has identified a rice line that can quickly eliminate Tos17 and pointed out that there is indeed a mechanism for removing Tos17 in the rice genome [91]. They speculate that mechanisms such as intra-element recombination may have caused element loss, resulting in internal deletion of reverse transcripts, or there may be an unknown mechanism in some rice genotypes that leads to significant loss of TE. This also poses certain difficulties for the study of Tos17 insertion mutations.

## 3. Strategies for Genotype-Phenotype Association

When inducing or inserting mutations to obtain the desired agronomic trait, we also need to understand the target gene information that controls the trait to ensure that the trait can be stably inherited. Therefore, breeders began to shift their focus to quickly and efficiently identify target genes, thus developing a series of identification methods, such as BSA, QTL mapping GWAS, molecular markers, etc. These methods have gradually become auxiliary means in various breeding stages.

### 3.1. Bi-Parental Mapping

The creation of bi-parental populations, such as F_2_, back-crosses (BC), doubled haploids (DH), recombinant inbred lines (RIL), near-isogenic lines (NIL), and molecular markers, is the first and most crucial stage in the bi-parental mapping approach. The general steps involved in bi-parental mapping are as follows: (1) gathering parental genotypes that differ for desired traits; (2) choosing molecular markers that differentiate the two parents, such as SSR and SNP; (3) creating a mapping population; (4) genotyping and phenotyping the mapping population; and (5) identifying QTL using an appropriate statistical technique. It is also reported that allele frequencies, QTL effects, and the kind and size of the mapping population all have an impact on the detection power of QTLs. The primary drawback of bi-parental mapping is that QTL localization can be restricted to intervals of 10–20 cM since very few recombination events occur throughout population development. Furthermore, phenotypic diversity of both parents is a prerequisite for QTL discovery in bi-parental populations, and this may explain only a small portion of the species’ genetic variance [92].

### 3.2. Multi-Parent Mapping

The drawbacks of bi-parental mapping have been addressed with the implementation of a multi-parent mapping technique. The genetic diversity of several parents produces a population with significant phenotypic variation, making it appropriate for high-resolution QTL mapping. Two multi-parent mapping experimental designs that are becoming increasingly common are multi-parent advanced generation intercrosses (MAGIC) and nested association mapping (NAM).

Yu et al. proposed NAM for analyzing the genetic architecture of maize flowering time [93]. A total of 5000 RILs from 25 families, with 200 RILs from each family, were produced by crossing 25 different maize inbred lines to the B73 inbred, which was selected as a reference line. The NAM population provides high resolution and power for QTL detection since it is a composite of multiple high-resolution bi-parental populations in one large population. Large-scale genetic mapping for several essential features, such as disease resistance and leaf architecture, has been conducted in maize using the NAM approach [94,95]. A. thaliana was used to create the first MAGIC population in crops [96]. This population comprised 527 lines that were produced by crossing a diverse panel of 19 founders. The QTL for hectoliter weight and plant height in wheat has been identified using MAGIC mapping [97]. For QTL mapping and varietal development in rice, MAGIC populations, including several *indica* and *japonica* rice parents, have been created [98]. MAGIC populations, in contrast to other multi-parent populations, involve mixing several inbred progenitors over several generations before creating inbred lines, which significantly increases the accuracy of QTL identification. MAGIC mapping offers excellent chances to analyze intricate features and enhance breeding populations.

### 3.3. Bulked Segregant Analysis

With the development of high-throughput sequencing technology, bulked segregant analysis (BSA) efficiency in locating target genes has also improved [99]. BSA, that is, mixed pool segregation analysis, firstly selects parents with relative traits to construct a segregation population, selects a certain number of individuals with large phenotypic differences to construct mixed pools, and realizes QTL mapping based on the differences of DNA molecular markers between the two mixed pools. Generally, recombinant inbred lines or near-isogenic lines will be selected as recombinant inbred lines for BSA analysis [100]. The MutMap method can also be used to study the relationship between character differences and the mutation site of mutagenic individuals [101]. The mutant was backcrossed with the homozygous parent, and the extreme character individuals were selected from the F_2_ or BC1 segregation population to construct a mixed pool. After that, high-throughput sequencing and correlation analysis were conducted [102]. It can be compared with the reference genome, and functional annotation can be made for genes in the location area. BSA overcomes the limitation that many crops have difficulty obtaining near-isogenic lines, saving time and effort. It is a convenient method for gene marker mapping, and breeders will widely use it. The genome and transcriptome levels were identified using F_2_, F_3,_ and F_4_ populations, and two rice homologous fertility-related genes were successfully identified in polyploid rice [103,104,105]. Nowadays, BSA can be analyzed at DNA, RNA, and protein levels, but the technology at the protein level is still limited [100].

### 3.4. Genome-Wide Association Study

**Figure 2:**
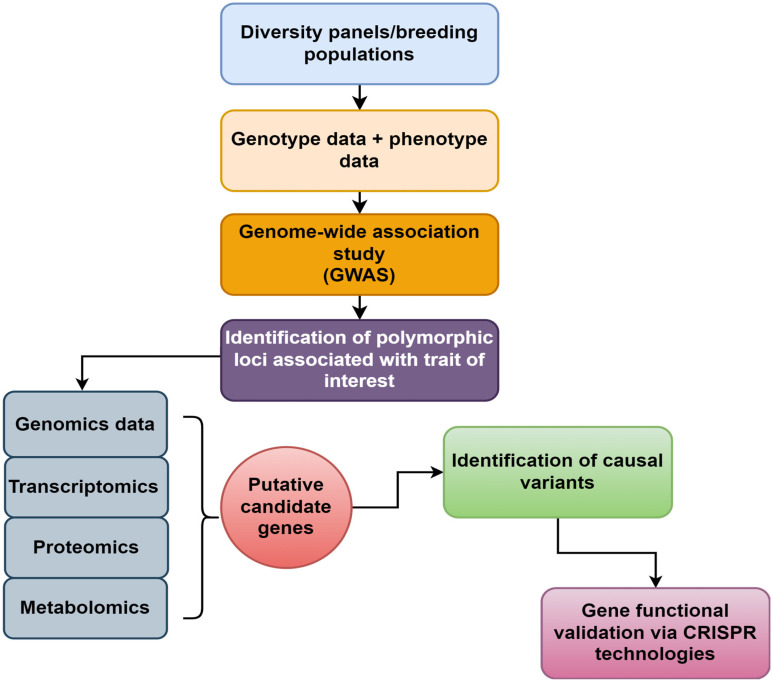
Scheme for identification of putative candidate genes applying genome-wide association study approach and their validation using CRISPR technologies.

A GWAS (Genome-wide association study) is used to detect the genetic variation polymorphism of from hundreds to tens of thousands of individuals within the whole genome. The larger the population, the more accurate the result [106]. The general population size is greater than 200 materials [107]. However, in population selection, it is necessary to ensure that genetic and phenotypic variations are abundant and that there can be no reproductive isolation in population structural differentiation [108]. Genotyping and haplotype identification were carried out with a large amount of data, and QTL analysis was conducted with the expression amount of transcriptome sequencing. Finally, genes related to character variation were mined [109]. The SNP locus identified by GWAS may not be the most significant and falls in the non-coding region. In addition, the accuracy of phenotype statistics is insufficient, or the phenotype is more affected by the environment, which has hindered the understanding of the relationship between the mutation locus and the phenotype [110,111]. It is, therefore, important to validate the identified locus/gene to be used in future marker-assisted selection programs in crop improvement. Conventional GWAS can carry out high-density genotyping for a large number of individuals. Recently, XP-GWAS has been developed that does not need to carry out genotyping for a large number of individuals. It can analyze extreme phenotypes and find the relationship between genetic variation and target traits [112]. In the BC-NAM (Backcross Nested Association Mapping) of sorghum, GWAS analysis was used to identify the genetic loci related to grain size [113]. GWAS has a higher resolution, a wide range of material sources, and rich variations that can be captured, and breeders do not need to build a recombinant inbred line, thus saving time. Numerous genes and QTL regions for different phenotypes have been discovered by GWAS over the past few decades. Many of these data are accessible through public databases, including the GrainGenes database for wheat and oats (https://wheat.pw.usda.gov/GG3/, accessed on 1 August 2024), MaizeGDB for maize (https://maizegdb.org/) (accessed on 1 August 2024), and QTARO ([1]; http://qtaro.abr.affrc.go.jp/) (accessed on 1 August 2024) and Gramene (https://www.gramene.org) (accessed on 1 August 2024) for rice. New approaches to combine genetic mapping, genomics, and precision phenotyping to find candidate genes underlying important QTLs of interest must be investigated since mapped QTLs offer a direct link to breeding-relevant genetic loci. Subsequently, these candidate genes may enable more sophisticated breeding methods like CRISPR-Cas gene editing (Figure 2). Key elements for carrying out GWAS have been explained as follows:

#### 3.4.1. Populations Selection for GWAS 

GWASs frequently require large sample sizes to uncover reproducible genome-wide significant associations. Power calculations in software programs can be used to establish the desired sample size. When the trait of interest is quantitative, research designs may include quantitative measures on the entire study sample or cases and controls when the trait is dichotomous. The necessary sample size determines the data source and study methodology for a GWAS. It takes a lot of effort and money to assemble data sets big enough to run a powerful GWAS for a complex trait. However, some GWASs use pre-existing resources, as many outstanding public sites offer access to large cohorts containing genotypic and phenotypic data [114].

#### 3.4.2. Genotyping/Population Structure

The population structure of the studied individuals is ascertained through the process of genome sequencing. Characterizing the domestication history and genetic relatedness of the individuals under study requires knowledge about population structure. Individuals are usually genotyped using next-generation sequencing techniques like WES or WGS, which also include rare variants or microarrays for common variations. Given the current cost of next-generation sequencing, microarray-based genotyping is the most popular method for acquiring genotypes for GWAS. It is generally advisable to genotype each individual cohort on the same genotyping platform in a consortium-led GWAS. In contrast, the choice of genotyping platform depends on several factors and is often dictated by the study’s objectives. The most ideal method would be WGS, as it can identify almost all variations in a genome. However, due to its low cost, microarrays and WES will likely lose ground to WGS technology over the next several years [114].

#### 3.4.3. Data Processing and Testing for Associations

Data on the genotyping and phenotype are included in the input files for a GWAS. Careful quality control is necessary after data input to ensure that a GWAS produces accurate results. Examples of actions include eliminating uncommon or monomorphic variants, screening for SNPs absent from a portion of the cohort, detecting and eliminating genotyping errors, and making sure phenotypes and genetic data match closely [114]. The biometrical model is the foundation of the notion of genetic association. Depending on whether the trait is continuous or binary, a GWAS typically uses either logistic regression models or linear regression models to test for associations. It should be noted that, in a GWAS, genotypes of genetic variants that are physically close to one another are not independent since these variations typically reside in linkage disequilibrium. This test dependency should also be taken into account throughout GWAS procedures [114].

#### 3.4.4. Accounting for False Discovery

To prevent false positives, a strict multiple-testing threshold is necessary when examining millions of associations between distinct genetic variations and a desired phenotype. A multiple testing correction is used to establish a threshold for significant associations. The Bonferroni correction with an alpha threshold of 0.05 is used as a conservative criterion at the genome-wide level. Additionally, the false discovery rate (FDR) with an alpha level of 0.05 is used to apply a less strict criterion for declaring significance. The control of population structure and genetic linkages in the various GWAS models was assessed using quantile–quantile (Q–Q) plots of the observed and expected p-values. The qqman R package is used to create these plots [115].

#### 3.4.5. Genome-Wide Association Meta-Analysis

GWAS can identify important genes that are associated with desirable traits. In addition to allowing for higher sample sizes, combining data from different populations is an appealing strategy for WGS-based GWAS since it increases haplotype diversity and breaks down LD blocks. Occasionally, multi-population GWASs are carried out by only adding the population as a fixed effect in the model; in other instances, multi-population meta-analysis techniques or more complex models are employed [116]. Regarding fine-mapping, a multi-population GWAS typically finds more connections and has narrower confidence intervals than an individual within-population GWAS. This is because it contains fewer variants.

### 3.5. Pan-Genome Analysis

**Figure 3:**
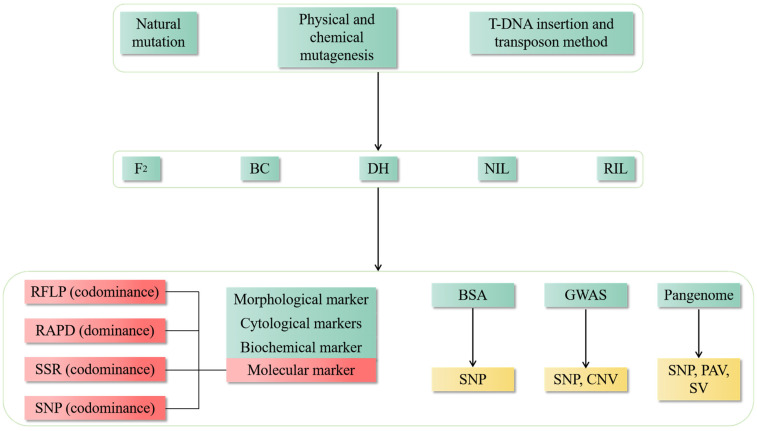
Forward genetics study.

The pangenome is the sum of all genes in a species, which can be divided into core genes and dispensable genes [117]. The core gene exists in the whole species, and the dispensable genome exists in single or multiple materials [118]. The pan-genome can mine SNP sites, but the sequencing depth determines the diversity of SNP sites. It can also provide new means for species evolution and wild resource mining. Pan-genome assembly methods include iterative assembly based on the reference genome, de novo assembly based on the reference genome, and de novo assembly not based on a reference genome [119,120,121,122]. It is used in Arabidopsis, maize, soybean, wheat, rice, and other crops [123,124,125,126,127]. As of 2017, 3000 rice genomes have been published, providing online genome sequence and annotation, PAV, and genome expression profiles [128]. There are a lot of germplasm resources in the world, and the functions of many genes are unknown. In view of the broad application of positive genetics, various markers and models are used to analyze the relationship between genetic variation and traits (Figure 3). The mining of gene function cannot stay in cytology or a single direction but in the whole process of genetic information transmission, such as the level of transcription and translation.

The green square represents the material population and experimental methods used in forward genetics research. The red square represents the commonly used mainstream marker. The yellow square represents a genetic variation that can be identified. F_2_: Filial generation 2; BC: Backcross, DH: Doubled Haploid, NIL: Near Isogenic Lines, RIL: Recombinant Inbred Lines, RFLP: Restriction Fragment Length Polymorphism, RAPD: Random Amplified Polymorphism DNA, SSR: Simple Sequence Repeats, SNP: Single Nucleotide Polymorphism, BSA: Bulk Segregant Analysis, GWAS: Genome-Wide Association Study, SNP: Single Nucleotide Polymorphism, CNV: Copy Number Variation, PAV: Presence/Absence Variation, SV: Structural Variations.

### 3.6. DNA Molecular Markers

Molecular marker-assisted breeding locates and selects the genotype of target traits by analyzing the genotypes of molecular markers closely linked to the target genes [57]. Detecting the existence of the target gene by molecular markers can help quickly and accurately select the target trait.

Genetic markers include morphological, cytological, biochemical, and molecular markers [129,130,131]. The morphological markers are the traits of genetic stability visible to the naked eye; cytological markers show variation in the number or morphology of chromosomes, and are displayed at the chromosome level; biochemical markers depend on storage proteins and isoenzymes, so the detectable sites are limited; molecular markers, which can directly reflect the changes in DNA levels, are currently commonly used markers [132].

RFLP (Restriction Fragment Length Polymorphism) is the first generation of DNA molecular marker technology. It changes the size of restriction endonuclease fragments according to the base mutation of restriction endonuclease sites in different varieties of genomes or the insertion and deletion of bases between restriction endonuclease sites, which can be detected by specific probe hybridization [133]. 

RAPD (Random Amplified Polymorphism DNA) is the second generation of molecular markers that involves PCR technology. It is amplified with random primers of 9–10 bp and then analyzed by gel electrophoresis imaging. Because there are few experimental steps and no probe is needed, it saves time and effort. However, it is generally a dominant marker with low repeatability and is prone to false positive or false negative results [134]. 

SSR (simple sequence repeat) markers are widely distributed in simple tandem repeats in eukaryotic genomics [135]. The repeated sequence is generally 1–5 bp, and primers are designed according to the specific sequence on the side of SSR for amplification. Polyacrylamide gel electrophoresis imaging is used to compare the relative mobility of the bands and the polymorphism of SSR loci [136]. In 1986, Ali [137] et al. first designed oligonucleotide probes that are specifically different from GATA’s simple repeated sequence mutation for human fingerprint analysis. In 1989, Litt [138] et al. amplified the microsatellite sequence when studying the allele on the SSR site of the human genome and named it “Microsatellite”. The emergence and wide use of SSR technology has laid a solid foundation for the research of crop genetic diversity in crop genetics and breeding, the construction of linkage genetic maps, and molecular marker-assisted selection ([139,140]. However, the development process of SSR primers is cumbersome and expensive.

SNP (single nucleotide polymorphism) is sequence diversity caused by single nucleotide variation, with high frequency, stable inheritance, and easy genotyping [141]. Differences in nucleotides between individuals make each SNP in a single copy of DNA a potentially useful marker, so SNP markers have a potential that cannot be underestimated [142]. Compared to other markers, high-throughput SNP genotyping allows for large numbers of markers and the rapid processing of large populations, multiple genotyping systems to meet different needs, and the ability to directly call alleles generated from double alleles and store a database of SNP markers [143]. Because of the large number and wide distribution of SNPs, which are usually adjacent to some disease genes and have a high correlation with susceptible disease genes, SNPs are mostly used in the research of the genetic anatomy of complex traits and diseases and gene recognition based on population [144].

An InDel refers to the insertion or deletion of a small piece of sequence at a particular position in the genome, usually less than 50 bp in length. Unlike SNPs, they are the insertion or deletion of DNA segments of different sizes in the genome, rather than a single base change. Their distribution frequency in the genome is also second only to SNP [145], and many of them occur in the gene, even in the exon region, promoter region, and other important positions. This kind of variation can often cause major changes in gene function, and an InDel is also a very important variation in genome structure. InDel molecular markers can design polymorphic primers according to the insertion or deletion of different-sized nucleotide fragments on the same genome site between different individuals [146]. InDel molecular markers have been used in the genetic analysis of plant and animal populations, molecularly assisted breeding, and other research because of their stability, high polymorphism, and simple typing system [147,148]. The predecessors also designed the “cumulative InDel” [149] model to make the evolutionary comparison faster and more accurate. However, the genetic information carried by InDel is limited, and, to achieve sufficiently high resolution, it is necessary to combine more InDel loci, which increases the technical difficulty of concentrating many loci in the same multiplex amplification system.

Cleavage amplified polymorphic sequences (CAPSs) are a kind of co-dominant molecular marker. Firstly, specific PCR primers (19~27 bp) are designed from DNA sequences of known sites to amplify a DNA fragment at this site, and then the amplified bands are cut by a specific restriction enzyme and analyzed by RFLP [150]. At present, this technology has been applied to pepper [151], sweet potato [152], tea tree [153], and other crops.

In order to solve the problems that RAPD technology takes a long time, can not be used as a hybridization probe, and is sensitive to the change of reaction conditions, Paran and Michelmore [154] developed the SCAR (Sequenced Amplified Specific Sequences Amplification) marker. Recovering the specific marker fragment from the gel, cloning and sequencing, and designing a pair of specific primers (18–24 bases) according to its base sequence, or sequencing the end of the RAPD marker, and adding about 14 bases to the end of the 10-base primer used in the original RAPD to become a specific primer complementary to the end of the original RAPD fragment. Compared with RAPD markers, SCAR results are stable and reproducible. In 2020, Quoc [155] et al. developed SCAR markers related to the pathogenicity of Magnaporthe grisea, which improved the control efficiency of rice blast. Ambreetha and Balachandar [156] reported that promoting the application of SCAR marker technology can quickly and accurately identify the pathogen level of microorganisms, thus avoiding the import and export of infectious plant materials.

In 2001, Li and C.F. Quiros [157] developed a simple marker technique called SRAP (sequence-related amplified polymorphism) which amplified the specific region of ORFs (Open reading frames) of the gene through a unique double primer design, and the upstream primer was 17 bp long, specifically amplifying the exon region. The downstream primer was 18 bp long, and the intron and promoter regions were specifically amplified. The marker was developed from Brassica crops, but it is widely used in garlic [158], prairie grass [159], tomato [160], and other crops.

Long-terminal repeat transposons, LTR-RTs, are one of the transposons with long terminal repeat sequences on both sides. Because of its large number and long length, LTR has high genomic content in plant transposons. Based on the genetic properties of the LTR retrotransposon, several molecular markers based on the LTR retrotransposon have been developed and are in widespread use. For example, Kalendar [161] et al. developed a new PCR-based marker system in 2010, which is used to rapidly separate the end and full-length elements of retrotransposons and has become an ideal choice for other species with underdeveloped marker systems. The iPBS molecular marker technology provides an effective tool for plant genomics research. At present, the crops that use this technology system for classification identification and assisted breeding include wheat [162], potato [163], cowpea [164], grape [165], etc. At present, iPBS molecular marker technology is widely used in plant germplasm identification, genetic structure, and genetic diversity analysis. With the development of sequencing technology, high-throughput sequencing will stand out among various technologies with its high efficiency and cost-effectiveness and gradually become the main research means of genetics and genomics. If the high-throughput sequencing platform can be combined with iPBS technology to screen out a large number of LTR sequences with high insertion polymorphism, more efficient markers can be developed for co-analysis and high-throughput sequencing. iPBS will then become a critical technology for biological research. Some other markers were also being used for QTL/gene mapping, such as sequence-tagged sites (STSs), expressed sequence tags (ESTs), diversity arrays technology (DArT), and high-resolution melting (HRM) analysis.

Genomic selection (GS) and speed breeding approaches utilize molecular markers as potent tools to enhance the effectiveness of breeding operations. These techniques enable breeders to make more precise and expedited selections for desired traits using genetic information instead of only observable characteristics. Examples of these technologies include marker-assisted selection (MAS), reducing breeding cycles (beneficial for perennial crops and fruit trees), improving genetic gain, predicting breeding values (such as milk production in cows), integrating disease resistance genes in cross-breeding, enhancing double haploid production, facilitating rapid gene introgression from wild relatives, using genomic selection in complex traits, assessing diversity, and conserving germplasm [131,166]. In brief, molecular markers play a crucial role in genomic selection by enabling more accurate, effective, and expedited selection procedures for desirable characteristics. This results in increased genetic accumulation and improved breeding programs in diverse species. The segments associated with the target trait were analyzed through various gene association analyses and mapping and further precise localization through molecular markers. Finally, the specific point of a certain gene continues to be studied.

## 4. Genetic Information Transfer of mRNA

The transmission of genetic information follows the central dogma [167], which is that it is transcribed from DNA into RNA and translated into protein. Changes in genetic information at different levels can affect gene function [168]. During each period of genetic information transmission, various epigenetic modifications are also used to adapt to changes in the environment [169]. Therefore, studying the genetic mechanism of crops at different levels of regulation makes the conclusion more reliable. 

The regulation of eukaryotic transcript levels is affected by multiple factors [170]. The regulation of transcriptional activation of eukaryotic genes has three elements: DNA regulatory sequences [171], regulatory proteins [172], and RNA polymerases [173]. Cis-acting elements [174,175] are eukaryotic regulatory elements, including promoters, enhancers, and silencers [176]. The activity of RNA polymerase is altered through protein–protein and DNA–protein interactions, thereby regulating transcription [173]. Enhancers generally bind to transcriptional activators and act on the transcription initiation complex to enhance the activity of RNA polymerase, thereby promoting transcription [177,178]. Silencers generally bind to transcriptional repressors for negative regulation and inhibit transcription [179]. Transacting factors are regulatory proteins that affect transcription [180], including basic and specific transcription factors. Specific transcription factors can bind to enhancers or silencers to regulate transcription [181]. Mutants with loss of function of C-repeat binding factors (CBFs) are susceptible to cold stress [182]. Under various abiotic stresses, there will be various transcriptional responses, such as salt stress, cold stress, high temperature, drought, etc. [183]. Therefore, regulation at the transcriptional level is very important.

Post-transcriptional regulation in eukaryotes is because transcription cannot directly generate mature mRNA but requires the modification and processing of its precursor heterogeneous RNA (hnRNA), which can improve the stability of the transcript [184]. The “cap” at the 5’ end is 7-methylguanine-triphosphate nucleoside, which is modified when the hnRNA is generated to 25-30 nucleotides. The modification at the 5’ end can be recognized by the small ribosomal subunit, which promotes the binding of mRNA to the ribosome and ensures that its translation starts from the initiation codon. It is also possible to block mRNA at the 5’ end, protecting it from degradation by exonucleases. It can also help mRNA to cross the nuclear membrane and be transported into the cytoplasm [185,186]. The “tail” at the 3’ end is polyadenylation (polyA), which is used to maintain the activity of the mRNA as a translation template and to increase the stability of its mRNA itself. For example, the 3’ ends of plant microRNAs have different types of tailing modifications to accelerate their degradation [187]. Introns are spliced after transcription because exons are coding sequences and introns are non-coding sequences. First, adjacent exons come close to each other, and the introns form lariat RNA. Then, through two transesterification reactions, the introns are excised, and the exons are connected to each other to generate mature mRNA. In the cytoplasm, many RNA-binding proteins perform post-transcriptional regulatory functions [188]. For example, maizeRNA-binding MA16 protein, Z. maize DEAD box RNA helicase protein 1 (ZmDRH1), and fibrillin form a complex involved in ribosome RNA (rRNA) metabolism [189]. In conclusion, many genes respond to different environmental stresses during post-transcriptional regulation.

Factors associated with translational levels include the eukaryotic translation initiation factor (eIF), which controls protein translation initiation in vivo, selective translation of mRNA, and mRNA longevity [190]. The degree of phosphorylation of initiation factors, the length of the untranslated region at the 3’ end associated with mRNA lifespan, RNA editing, and RNA interference all regulate the level of gene translation [191]. General control nonderepressible 2 (GCN2) plays a pervasive role in alleviating cellular stress by directly binding to uncharged tRNAs and phosphorylating their target eukaryotic initiation factor 2alpha (eIF2α). AtGCN2 loss-of-function mutants exhibit increased resistance to necrotrophic pathogens [192]. Additionally, herbicides [193], UV light, cold stress [194], amino acid and purine starvations, wounding [195], cadmium, oxidative stress, etc. [196], can modulate translation processes by affecting GCN changes. Loss of function of the potato translation initiation factor eIF4E1 can reduce the growth of potato virus YPa36 and improve virulence-induced symptoms without affecting the homeostasis of mRNA levels of other genes in the family [197].

Various protein post-translational modifications (PTMs) increase the functional diversity of the proteome by covalently adding functional groups or proteins, proteolytic cleavage of regulatory subunits, or degradation of whole proteins [198]. Modifications, including phosphorylation [199], glycosylation [200,201], ubiquitination [202], nitrosylation [203], methylation [204], acetylation, lipidation, proteolysis, etc., affect various aspects of normal cell biology, stress resistance, and tolerance [205]. The ubiquitin E3 ligase APIP6/RIP1 can degrade multiple susceptible proteins, resulting in broad-spectrum resistance (BSR) [206]. Histone acetylation, phosphorylation, and ubiquitination are thought to activate gene expression, and biotinylation inhibits gene expression [207]. Phosphorylation and ubiquitination regulate ABA signaling, respectively [208]. N-glycosylated proteins in the Golgi apparatus are associated with plant salt tolerance [209]. A heat-inducible dCas9 to target a JUMONJI (JMJ) (dCas9-JMJ) induced targeting ASCOBATE PEROXIDASE2 (APX2) can effectively reduce the methylation level of histone H3 lysine 4 (H3K4), which is involved in the heat stress response [210]. Histone deacetylase (HDA19) regulates the phosphate starvation response [211]. Therefore, exploring protein post-translational modifications is crucial in research.

**Figure 4:**
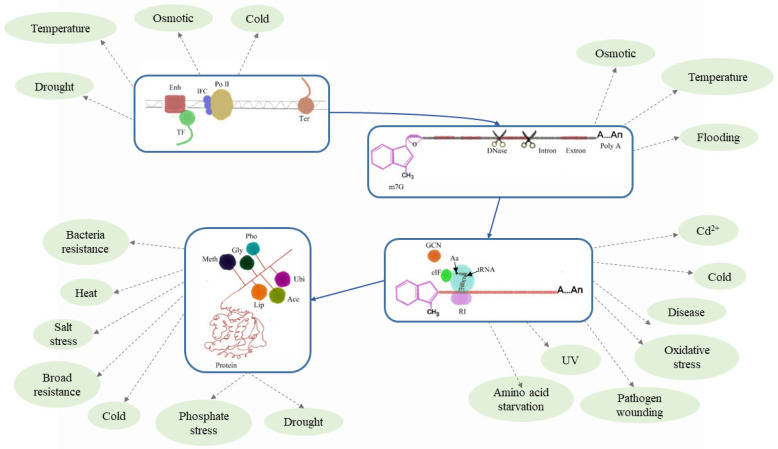
The regulation or modification involved in the central dogma process. A slight change in each link will affect the changes in gene function. Enh: Enhancer; TF: Transcription factor; IFC: Initiation factor coactivator; Po II: RNA polymerase II; mG7: 7-Methylguanine-nucleoside triphosphate; DNase: Deoxyribonuclease; Poly A: Polyadenylic acid; GCN: General control nonderepressible; Eif: Eukaryotic translation initiation factor; Aa: Amino acid; tRNA: Transfer RNA; RI: Ribosome; Meth: Methylation; Gly: Glycosylation; Pho: Phosphorylation; Ubi: Ubiquitination; Ace: Acetylation; Lip: Lipidation.

Multiple factors regulate the complexity of genetic information transmission and also respond to various environmental stresses (Figure 4). In breeding, it is particularly important to pay attention to the regulation of the whole process and not to focus on a single process.

## 5. Gene Editing

Improving genetic characteristics and cultivating new varieties of excellent plants are the goals of breeding [5]. The breeding methods can be divided into conventional breeding and unconventional breeding [6]. Hybrid breeding and distant hybridization, which belong to conventional breeding, are more traditional, while non-conventional breeding can be divided into mutation breeding, in vitro culture, and molecular breeding. Molecular breeding is a relatively fast and efficient breeding method at this stage [7]. Gene editing is an emerging molecular research technology that is widely used in animals and plants [212,213,214] and has greatly promoted modern breeding [3,215]. The first nucleases used in gene editing are ZFNs [216] and TALENs [217], and then comes CRISPR [218,219] (Table 1). ZFNs and TALENs are endonucleases that break DNA double strands and are guided by proteins. 

ZFN is an artificially modified endonuclease that consists of a DNA recognition sequence composed of zinc finger proteins in series and a non-specific endonuclease. However, due to the low specificity of its zinc finger motif at the same time as the target sequence, it is necessary to the specific recognition of a specific target requires the construction of a huge expression library, which makes it difficult for us to find suitable targets. TALEN, as a protein-guided endonuclease similar to ZFN, has high target specificity and is more convenient for target design. Both bind to the target in the form of a dimer, which activates the cleavage domain of the endonuclease and breaks the double bond.

Until the advent of CRISPR/Cas9 [220], gene editing technology developed rapidly. Instead of protein–DNA binding, this endonuclease uses RNA-DNA binding to direct nuclease activity and does not require dimerization to function. Since then, dCas9 [221,222], Cas13 [223], dCas13 [224], and Cas12/13 [225,226] have been developed. Cas systems include gene knock-down, out, in, activation, evolution, in vivo chromatin imaging, epigenetic modification, base editing, and lineage tracing systems [227]. In prokaryotes, CRISPR (clustered regularly interspaced short palindromic repeats)/Cas functions as part of the adaptive immune system: invading phage and plasmid DNA is targeted by complementary CRISPR RNAs (crRNAs) bound to CRISPR-associated endonucleases [218]. The repair mechanisms after double-strand breaks are also different. Three are currently known. The first is NHEJ [228,229], non-homologous end joining. The repair mechanism does not require any template repair protein to pull the two ends of the break directly and join them directly under ligase. Then, there is HDR [230], homologous recombination repair, which can only occur when there are DNA fragments homologous to the damaged DNA in the nucleus. The CRISPR/Cas system was first introduced into human 293FT cells [227] and has since been widely used in plants.

The safety of genetically modified crops has always been a concern, and whether the insertion of exogenous fragments will cause harm to the human body has not been well explained [128,231]. However, transgenic crops are an effective means to study gene function and can also effectively improve plant disease resistance [232], drought tolerance [233], salt tolerance [234], and other effects in Arabidopsis [235], soybean [236,237], maize [238], rice [239,240], wheat [241], cotton [242], tobacco, potato [243], tomato [244], and other plants. However, different countries have different control conditions for genetically modified crops. Hence, identifying whether it is a genetically modified plant is also very important, and a simple and effective identification method is also derived [245]. The sooner the edited and GMO-free seeds are obtained at an earlier generation, the easier it will be for subsequent studies. According to the law of Mendelian inheritance, the transgenic seed progeny of the T0 generation can obtain 1/4 of the non-transgenic plants. Researchers use some special strategies to try to obtain GMO-free seeds in the T0 generation. For example, by the method of transient transformation editing, no antibiotic selection is used after transient transformation editing so that it can be edited in plant cells without being transferred into the plant genome [246,247]. The promoter of REG2, expressed explicitly in early rice embryos and the male gametophyte-specific lethal protein CMS2, is constructed into a transgenic vector, which can ensure that the progeny seeds are all non-transgenic seeds. This is because the CMS2 protein can destroy mitochondrial function, leading to male sterility [240,248,249]. A fluorescent marker for m-Cherry can also be added to transgenic elements, using red fluorescence to identify segregated seeds [250]. In addition, the GFP fluorescent protein can be linked with the Cas9 protein without adding a promoter, so these two proteins can be expressed simultaneously to achieve the purpose [251]. However, gene editing is neither efficient nor 100%, and the editing efficiency and difficulty vary among different crop species [252,253]. For example, it is also regulated at the post-transcriptional level, and defects in the post-transcriptional gene silencing pathway can significantly elevate Cas9 and sgRNA transcript levels, resulting in higher mutation frequencies than in wild-type controls [254]. In conclusion, gene editing technology has played a revolutionary role in plant breeding.

## 6. Artificial Intelligence (AI) Breeding

The rapid development of artificial intelligence technology and modern advanced technology has led crop breeding to a new stage. Machine learning (ML) is a sub-class of artificial intelligence (AI), and data can be analyzed as supervised (targeted values) or unsupervised (non-targeted values of outcome). Both of them are prevalent extensively in plant breeding and other disciplines of plant sciences [255]. With the integration of AI technologies, breeders can analyze and interpret vast amounts of data generated from various sources, including genotypic and phenotypic data, to make informed decisions in the breeding process. 

High-throughput phenotyping techniques involve the use of digital data acquired through different techniques both in the field, in a greenhouse, or under laboratory conditions, with the aid of sensors, data collection and computation, and computer software [256]. The use of AI has revolutionized plant phenotyping by using digital images followed by computing to find out the specific attributes needed to recognize a specific target or object. ML uses different tools and approaches to collect data related to phenotypes, extracting the results using the classification of the data and identifying new features and patterns to predict novel trends [257]. The use of AI-assisted high-throughput phenotyping systems in different plants with desired targets has been employed for growth stages in wheat and maize [258], oilseed crops for semantic segmentation of the crops and weeds [259], and improved plant production [260].

Cyberinfrastructure (CI) is a platform for researchers to store data and use computing systems using high-performance networks [244] to integrate AI in other domains of phenotyping. Open-source devices and tools are extensively used in phenomics and enable the private sector to invest in manufacturing and distributing phenotyping technologies alongside government-funded agricultural institutions [261]. Field-based phenotyping (FBP) is the most significant and adaptive technology for the precise depiction of traits. It involves the use of multiple sensors [256] carried out by vehicles, unmanned aircraft, or robots [261].

Another application of AI in plant breeding is the use of computer vision technologies for plant phenotyping, which involves analyzing several plant characteristics such as plant architecture, leaf morphology, and color. The use of deep learning (DL) algorithms, which involve computer vision technologies such as image analysis [262], enables the automatic measurement of these traits, reducing the time and labor required for manual phenotyping. This also increases the accuracy and precision of the phenotypic data collected, resulting in more accurate predictions of plant traits. The use of DL has been employed successfully to solve complex biological problems in different disciplines of plant omics [263]. These include the determination of transcription factor DNA-binding specificity [264], gene expression [265], and a protein’s tertiary structure [266].

AI technologies have also been used to identify genomic regions associated with specific traits, known as quantitative trait loci (QTL) mapping. This involves the analysis of genotypic and phenotypic data to identify the genetic basis of traits. AI-based QTL mapping approaches, such as Bayesian networks [267], are effective in identifying QTLs associated with complex traits. The use of such AI algorithms enables us to understand the genetic basis of these traits and to develop plant varieties with more elite traits.

### 6.1. Beneficial Aspects of AI Technologies to Overcome the Phenomics Bottlenecks to Improve Crop Breeding

**Figure 5:**
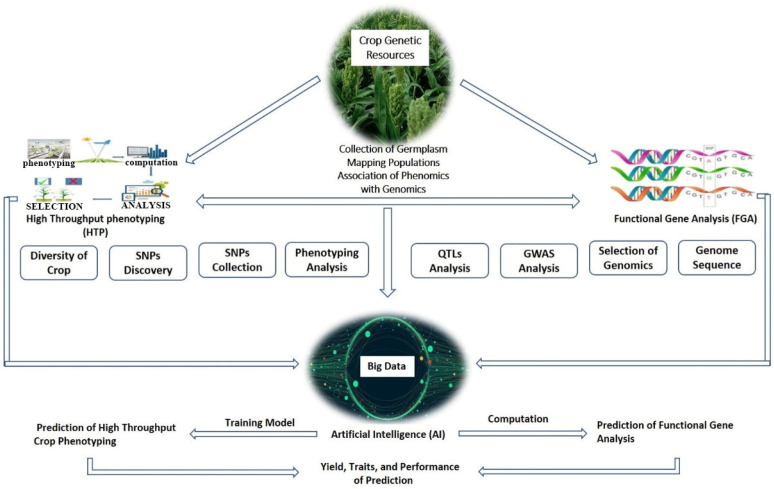
Graphical presentation of AI application as a powerful tool for predicting HTP of crop and FGA in modern crop breeding.

Recent advances in plant phenomics have opened a new door for precision breeding (Figure 5). This development can be attributed to the rise in new technology invention and accessibility that enables high-throughput phenotyping of complicated plant traits. The application of AI in numerous scientific domains has skyrocketed in recent years. DL, ML, and computer vision components of AI have been successfully incorporated into non-invasive imaging techniques [268]. This integration steadily increases the effectiveness of data collection and analysis by using ML for robust picture analysis. Three essential aspects of phenomic data management involve the use of AI: algorithms and programs to transform sensory data into phenotypic information; model development to comprehend genotype-phenotype association with environmental factors; and database management to facilitate the exchange of data and resources [269].

Although field phenotyping is a fundamental practical need for crop breeding, high-throughput phenotyping in the field still remains behind the presently convenient indoor phenotypic facilities. Therefore, more work needs to be performed to develop these facilities and investigate the practical applications of phenomics. AI technologies require a sizable amount of data from multiple sources in order to boost their accuracy. This creates the opportunity for further investment in field data collection, the modification of existing technologies, and the use of readily accessible AI-adaptable technology, like smartphones, to increase the quantity and quality of data collected [270]. The accessibility with which their cameras and sensors may be employed suggests that smartphones will find tremendous value in agriculture as they become more widely used as consumer products [261]. On smartphones, effective signal processing must work around limitations such as short battery life, constrained CPU capacity, and constrained bandwidth. The amount of data that may be obtained by using citizen science with professional researchers is potentially greater [270]. The major goals of employing these methods and technologies are to provide the infrastructure for monitoring the development of plants throughout the growing season and to simplify data storage, analysis, and application to AI algorithms [271].

### 6.2. Role of AI in Automation and Digital Agriculture

Automation in data collection and analysis significantly accelerates genetic gains in plant breeding and genetic research. Automated systems collect vast amounts of data, such as phenotypic and genotypic information, more accurately and at a faster pace. This rapid data acquisition allows researchers to make more informed selections and decisions in breeding programs, shortening the breeding cycle and increasing the pace of desirable traits being incorporated into new plant varieties [272]. Real-time data analysis enhances genetic gains by allowing dynamic decision-making. AI-powered image analysis and sensor technologies can precisely measure phenotypic traits, ensuring only the best-performing plants are selected. AI can also model breeding techniques, assess historical data, and optimize breeding program designs through statistical and machine-learning models. Scalability allows for the automation of large-scale breeding programs, leading to more robust genetic insights and faster dissemination of improved varieties across different regions and climates [273]. The integration of UAVs and satellite imagery in plant breeding is revolutionizing the field by enabling high-throughput phenotyping, enhancing genetic diversity assessments, and improving early variety selection. When combined with AI, this significantly enhances the selection of the best-performing varieties at early breeding stages [274,275].

Advanced UAVs collect high-resolution data from agricultural fields, enabling breeders to assess genetic diversity and respond to environmental stressors. These data help select plants that are more resilient to stresses, contributing to robust crop varieties. UAVs reduce human error, improve selection criteria reliability, and optimize time and resources by reducing labor and costs. Rapid data collection speeds up breeding cycles, enabling multiple cycles within shorter periods [276]. High-throughput phenotyping (HTP) using UAVs and drones to collect detailed data on plant health, stress levels, and nutrient content is revolutionizing modern breeding programs. It uses thermal imaging to detect plant stress and phenotyping at the early stages of breeding [277]. UAV data can also be used for disease and pest surveillance, assessing environmental stress, identifying nutrient deficiencies, aiding in the selection of resistant varieties, and improving nutrient use efficiency [278]. Satellite imagery provides extensive spatial coverage for monitoring crop performance across diverse landscapes, aiding in climate impact studies, soil and terrain mapping, and selecting climate-resilient crops [279]. It can be combined with UAVs and drones for multi-scale data integration, providing a comprehensive view of plant performance from plot to regional scales. Advancements in data processing include cloud computing for efficient handling of large datasets, real-time analysis for breeding programs, and specialized software tools for in-depth analysis and user-friendly interfaces [280].

### 6.3. Beneficial Aspects of AI in Gene Function Analysis to Improve Crop Breeding

Massive amounts of data have been produced in recent decades as a result of the quick development of high-throughput technology in the biological sciences. The term “omics” is frequently used to refer to fields that aim to gather and analyze huge amounts of biological data. It stands for the entire amount of DNA that is present in each cell of an organism, with the added connotation of being open to complex problems [260]. Data evaluated from omics approaches have grown too massive and complex to be visually or statistically analyzed. AI has multidisciplinary uses in plant genomics and will likely have more in the future for comprehensive genome investigation. For various bioinformatics analyses, including cis-regulatory element identification, protein-coding gene identification, protein–protein interaction, gene expression, plant phenotype predictions, metabolic pathways, gene ontology, subcellular location, and genomic prediction, a variety of ML tools and algorithms can be utilized [281]. AI will probably be utilized in the not-too-distant future to handle a number of plant genomics issues. AI algorithms could be applied to comparative genomic studies or the transfer of knowledge (mean gene) from a model plant to a specific field plant [282]. To anticipate gene functions or the multiple ways gene expression affects a trait, a variety of expressions or analyses of sequencing data might be considered [283]. All of this adds up to provide biologists with a more “relevant” depiction of their data as well as the capacity to incorporate it, allowing them to study their genomic data, evaluate and verify their hypotheses throughout the experimental cycle, and eventually enhance their research.

Cost-effectiveness is another benefit of these technologies. While the initial investment in UAVs and related technologies may be significant, the long-term cost savings are substantial. These technologies reduce the need for extensive manual labor and allow for more frequent monitoring of crops, leading to earlier detection of issues and more informed decision-making [284]. This ultimately leads to higher crop yields and reduced losses due to pests, diseases, or environmental factors. UAVs, drones, and satellite imagery enable breeders to collect large-scale phenotypic data quickly and accurately, which is critical for assessing various traits such as plant height, canopy structure, and stress responses. The high-resolution images and multispectral data provided by these technologies enable the detection of subtle differences in plant performance that are often invisible to the naked eye, resulting in a more precise selection of desirable traits, speeding up the breeding process, and reducing the need for manual labor. Future perspectives highlight the transformative potential of UAVs, drones, and satellite imagery in plant breeding, contributing to more efficient, sustainable, and resilient agricultural systems worldwide.

In conclusion, AI technologies have significantly impacted the field of plant breeding, and their applications have revolutionized how breeders approach the breeding process. The integration of AI technologies has enabled breeders to make informed decisions, reducing the time and resources needed to produce new plant varieties and improving the success rate of breeding programs. The future of plant breeding is likely to see even greater integration of AI technologies, resulting in even more advanced and efficient breeding programs.

## 7. Current Challenges and Future Perspectives

The transmission of genetic information of a gene from transcription to translation is dynamic, and different functions may occur at different levels of fluctuations. Suppose a gene’s entire process, from DNA replication to post-translational modification, is studied in detail. In that case, the gene’s function will be better understood and more accessible to breeders. Including the most breakthrough molecular research technology at present, the most widely used function of gene editing is to perform irregular mutations within dozens of clips. The way this mutation is performed is uncontrollable, and the mutation results need further testing. As technology develops, directional changes are also being studied, from one clip to another defined base, but the efficiency of this mutation has yet to be improved. Moreover, gene editing can screen mutants inserted by foreign aid fragments, which differs from the transgenic plants that breeders generally realize. Suppose a more effective and accurate technology for screening mutants without foreign aid fragment insertion can be found, and the efficiency of gene editing can be improved. In that case, applying gene editing technology will be a revolutionary change. Several genes can be edited in batches using transgenic technologies, such as precision insertion, substitution, and shearing of several bases in some genes, to monitor phenotypic changes and choose plants with diversity for future research. Random plant editing contributes significantly to the rise of biodiversity. Selective breeding was also carried out by screening organisms that did not include exogenous fragments, as well as crops that met the breeding goals. Selective breeding can help eliminate the detrimental effects of exogenous fragments on human health. As policy conditions become more flexible, biological engagement in gene editing will become increasingly greater. However, as the efficiency of gene editing increases, the genetic diversity of species may also be reduced. Because breeders may perform selective breeding on the basis of their own limited knowledge, some potentially interesting directions may be overlooked. 

Meanwhile, with the rapid development of new-generation gene sequencing and phenotype acquisition technologies, more and more genetic data, phenotype data, and environmental data are emerging in the field of crop breeding research, and the deep integration of artificial intelligence, information technology, and conventional breeding and biotechnology is promoting breeding from the traditional breeding process of hybrid breeding and field screening to a data-driven intelligent breeding technology system. In the new breeding system of the future, information technology and intelligent equipment technology will be applied in all aspects of breeding data acquisition, analysis, storage, management, and application, which will form a disruptive new breeding model in which computers assist or replace the work of the human brain and intelligent equipment replaces manual labor. Breeders will create genotype–phenotype–environment multidimensional extensive data-driven precision breeding programs, which will also lead to gene editing, synthetic biology, and other technological upgrades and superimposed integration, targeted and efficient improvement and breeding of new varieties, and promote precision, intelligence, and factory-based seed revolution. However, there are limitations to how breeders view the direction of plant development. Allowing crops to grow freely in nature is also an indelible part of research. Breeders have some responsibility for the direction of breeding but also need the mutual assistance of relevant institutions and policies.

## Figures and Tables

**Table 1:** Comparison of the three commonly used gene editing techniques.

	ZFN	TALEN	CRISPR/Cas
Combination mode	Protein–DNA	Protein–DNA	RNA–DNA
Identification length	(9 or 12 bp) × 2	(8–31 bp) × 2	20 bp + “NGG”
Cutting elements	FokI Protein	FokI Protein	Cas Protein
Composition	Zinc finger DNA binding domain + DNA cutting domain	N-terminal structural domain containing nuclear localization signal + Central structural domain of a typical tandem TALE repeat sequence + C-terminal structural domain with FokI	The commonly used Cas nuclease is Cas9 nuclease, which has two important nuclease structural domains RuvC and HNH.
Advantages	Easy design and high sequence specificity	High sequence-specific binding ability, high efficiency, and easy design	Precise targeting, low cytotoxicity, low cost and easy operation, low off-target rate
Disadvantages	Easy off-target, high cytotoxicity, high potential risk, low efficiency	High cytotoxicity, tedious assembly process, and high workload	PAM sequence must be present before the target site, low specificity

## Abbreviations

AI: Artificial intelligence; BSA: Bulked segregant analysis; CAPS: Cleavage amplified polymorphic sequences; GWAS: Genome-wide association study; InDel: Insertion-deletion; QTL: Quantitative trait loci; RAPD: Random amplified polymorphism DNA; RFLP: Restriction fragment length polymorphism; SNP: Single nucleotide polymorphism; SSR: Simple sequence repeats; MAS: Marker-assisted selection.
